# A novel disulfidptosis-related prognostic gene signature and experimental validation identify *ACTN4* as a novel therapeutic target in lung adenocarcinoma

**DOI:** 10.3233/CBM-230276

**Published:** 2024-02-12

**Authors:** Kai Xie, Bin Wang, Pei Pang, Guangbin Li, Qianqian Yang, Chen Fang, Wei Jiang, Yu Feng, Haitao Ma

**Affiliations:** 1 Department of Thoracic Surgery, The Fourth Affiliated Hospital of Soochow University, Suzhou, Jiangsu, China; 2 Department of Thoracic Surgery, The First Affiliated Hospital of Soochow University, Suzhou, Jiangsu, China; 3 Department of Pathology, The First Affiliated Hospital of Soochow University, Suzhou, Jiangsu, China

**Keywords:** Disulfidptosis, lung adenocarcinoma, ACTN4, immune infiltration, therapeutic target

## Abstract

**BACKGROUND:**

Lung adenocarcinoma (LUAD) is a prevalent form of malignancy globally. Disulfidptosis is novel programmed cell death pathway based on disulfide proteins, may have a positive impact on the development of LUAD treatment strategies.

**OBJECTIVE:**

To investigate the impact of disulfidptosis-related genes (DRGs) on the prognosis of LUAD, developed a risk model to facilitate the diagnosis and prognostication of patients. We also explored *ACTN4* (DRGs) as a new therapeutic biomarker for LUAD.

**METHODS:**

We investigated the expression patterns of DRGs in both LUAD and noncancerous tissues. To assess the prognostic value of the DRGs, we developed risk models through univariate Cox analysis and lasso regression. The expression and function of *ACTN4* was evaluated by qRT-PCR, immunohistochemistry and *in vitro* experiments. The TIMER examined the association between *ACTN4* expression and immune infiltration in LUAD.

**RESULTS:**

Ten differentially expressed DRGs were identified. And *ACTN4* was identified as potential risk factors through univariate Cox regression analysis (*P* <  0.05). *ACTN4* expression and riskscore were used to construct a risk model to predict overall survival in LUAD, and high-risk demonstrated a significantly higher mortality rate compared to the low-risk cohort. qRT-PCR and immunohistochemistry assays indicated *ACTN4* was upregulated in LUAD, and the upregulation was associated with clinicopathologic features. *In vitro* experiments showed the knockdown of *ACTN4* expression inhibited the proliferation in LUAD cells. The TIMER analysis demonstrated a correlation between the expression of *ACTN4* and the infiltration of diverse immune cells. Elevated *ACTN4* expression was associated with a reduction in memory B cell count. Additionally, the *ACTN4* expression was associated with m6A modification genes.

**CONCLUSIONS:**

Our study introduced a prognostic model based on DRGs, which could forecast the prognosis of patients with LUAD. The biomarker *ACTN4* exhibits promise for the diagnosis and management of LUAD, given its correlation with tumor immune infiltration and m6A modification.

## 1. Introduction

According to research, lung cancer is the primary cause of cancer-related mortality on a global scale [1]. Specifically, non-small cell lung cancer comprises 85% of all cases, with lung adenocarcinoma (LUAD) being the most common subtype [2]. While driver mutations, including *EGFR*, *KRAS*, *ALK*, and *TP53*, play a critical role in LUAD [3,4,5], only about 30% of patients benefit from targeted treatment [6]. Despite the implementation of diverse therapeutic approaches, including surgery, radiotherapy, chemotherapy, and immunotherapy, certain patients continue to encounter postoperative recurrence and metastasis [7,8]. Therefore, there exists a pressing necessity to devise dependable and efficacious prognostic biomarkers, construct risk prognostic models, and guide physicians in evaluating patients for individualized and optimal treatment. This is especially important given the rapid development of bioinformatics.

A recent study has identified a novel form of programmed cell death, termed disulfidptosis, which is reliant on disulfide proteins [9]. The current body of research indicates that the presence of disulfides is linked to changes in cellular redox state, which can induce the demise of neoplastic cells by modifying the conformation of cytoskeletal proteins. Consequently, this discovery may represent a significant advancement in the field of tumor therapy, and additional investigation and inquiry into the precise mechanism is warranted.

Disulfide metabolism pertains to the biochemical process through which disulfides are transformed into more stable compounds via diverse chemical reactions within the human body. Recent research has revealed that tumors exhibit anomalies in the expression and operation of enzymes responsible for disulfide metabolism, which could potentially be linked to the occurrence and treatment of cancer [10,11,12]. The aberrant expression and function of these disulfide-metabolizing enzymes may result in the accumulation and heightened toxicity of disulfides, thereby fostering the development and advancement of tumorigenesis. Moreover, the metabolism of disulfide bonds in cancer cells has been associated with various biological phenomena, including drug resistance, metastasis, and immune evasion, suggesting a potential correlation between disulfide-mediated apoptosis and tumor immune response [13,14]. Notably, *ACTN4*, a cytoskeletal protein family member, is responsible for binding to actin filaments to uphold cytoskeletal architecture and cellular morphology [15,16]. The migration of cancer cells within the extracellular matrix necessitates actin polymerization and rearrangement, thereby playing a pivotal role in cancer cell motility [17,18]. Numerous studies have indicated that the upregulation of *ACTN4* is commonly associated with an adverse prognosis, metastasis, and aggressive phenotype in various types of cancer [19,20,21]. High *ACTN4* expression has been recognized as a prognostic indicator of platinum-based therapy outcome in LUAD. In the cohort of patients exhibiting high *ACTN4* expression, the implementation of cisplatin-based adjuvant chemotherapy confers a significant clinical benefit in terms of overall survival [22,23]. A growing body of literature suggests that tumor immunotherapy and N6-methyladenosine (m6A) are crucial factors in the development of LUAD [24,25]. Nonetheless, the comprehension of *ACTN4* in LUAD, particularly the correlation between *ACTN4* and tumor immunotherapy and m6A modification, has received limited attention.

This research has developed a prognostic model utilizing DGRs to forecast the prognosis of LUAD patients. Additionally, the study has confirmed the role of *ACTN4* in A549 and PC9 cells. A multidimensional analysis was performed to assess the gene and functional network associated with the expression of *ACTN4* in LUAD, as well as to investigate the relationship between its expression and tumor immunity and m6A modification. The results of this investigation offer a theoretical foundation for identifying potential molecular mechanisms.

## 2. Materials and methods

### 2.1. Data acquisitions

RNA-seq data and clinicopathological parameters sourced from The Cancer Genome Atlas and Genotype Tissue Expression Database, encompassing 574 LUAD samples and 288 normal lung samples. The DRGs set were derived from the latest research [11,12].

### 2.2. Patient tissue samples

From 2019 to 2022, fifty paired LUAD tissues and adjacent non-tumor tissues were obtained from patients who were diagnosed with LUAD at the Thoracic Surgery Department of the First Affiliated Hospital of Soochow University. The clinical information pertaining to these patients was retrieved from their medical records. The Ethics Committee of the First Affiliated Hospital of Soochow University granted approval for this study, and informed consent was obtained from all participants. And the study conforms with The Code of Ethics of the World Medical Association (Declaration of Helsinki), printed in the British Medical Journal (18 July 1964).

### 2.3. Cell lines

The cell lines utilized in this study were procured from the Shanghai Institutes for Biological Sciences (China). Specifically, LUAD cells (NCI-H1975, PC9, and A549) were cultured in RPMI 1640 medium (KeyGene, Nanjing, China), and human bronchial epithelial cell (HBE) was cultured in DMEM medium (10% fetal bovine serum), respectively. All cells were maintained at 37^∘^C in a humidified incubator with 5% CO_2_.

### 2.4. RNA extraction and qRT-PCR

The process of extracting Total RNA from tissue samples or cells was conducted through the utilization of TRIzol reagent (Invitrogen, Carlsbad, CA, USA) in adherence to the manufacturer’s guidelines. Subsequently, reverse transcription was executed using the Prime Script RT kit (Takara, Nanjing, China), and qRT-PCR was carried out using the SYBR Select Master Mix kit (KeyGEN, Nanjing, China). Each qRT-PCR reaction was performed in triplicate as follows: step 1: denaturation at 95^∘^C for 10 min; step 2: 40 cycles of 95^∘^C for 15 s and 60^∘^C for 1 min. The primers presented in the Supplementary Table 1.

### 2.5. siRNA construction and cell transfection, cell proliferation assays

The siRNAs targeting *ACTN4* was produced from RiboBio (Guangzhou, China), and the procedures for cell transfection and cell proliferation assays were executed in accordance with previously established protocols [26]. The sequences presented in the Supplementary Table 1.

### 2.6. Immunohistochemistry (IHC)

IHC was conducted as described in refs. [26], and IHC was carried out using following antibodies: Anti-*ACTN4* antibody (#15145; Cell Signaling Technology, USA).

### 2.7. Identification of differentially expressed DRGs and prognostic genes

In this study, the “DESeq2” package was employed to detect DRGs that were differentially expressed (*P* <  0.05,  | log_2_ FC | < 2). Subsequently, the “string” database (https://string-db.org/) was utilized to map protein-protein interactions (PPIs). Furthermore, the “survival” package was utilized to conduct univariate Cox regression analysis.

### 2.8. Construction of prognostic model based on DRGs

The standardized mRNA data from TCGA-LUAD was utilized to establish the risk score, which was determined through Cox regression analysis. The coefficient of DRGs was represented by X, while the expression level of DRGs was represented by Y. Subsequently, LUAD patients were classified into high-risk and low-risk groups based on the median risk score, and OS was analyzed.

The prognostic performance of the models was assessed using receiver operating characteristic (ROC) curves generated by the “timeROC” package.

### 2.9. Construction of nomograms and calibration curves

Nomograms were generated utilizing the “RMS” package of R software to forecast individualized probabilities of survival, whereas calibration curves were constructed to prognosticate the survival rates of LUAD patients.

### 2.10. TIMER analysis

TIMER2 (http://timer.cistrome.org/) is an interactive web-based tool that utilizes the deconvolution method to infer the gene expression spectrum of tumor-infiltrating immune cells across various cancer types from the TCGA dataset [27].

### 2.11. Statistical analysis

The statistical analyses were performed using R software (version 4.1.1), and the experimental graphs were generated using GraphPad Prism software (version 8.0.). Unpaired t-tests were utilized for unpaired samples, while paired t-tests were employed for paired samples. The correlation of gene expression was evaluated using Spearman’s correlation. A statistical significance level of *P* <  0.05 was considered.

## 3. Results

### 3.1. Identification of differentially expressed DRGs in LUAD

**Figure 1.**
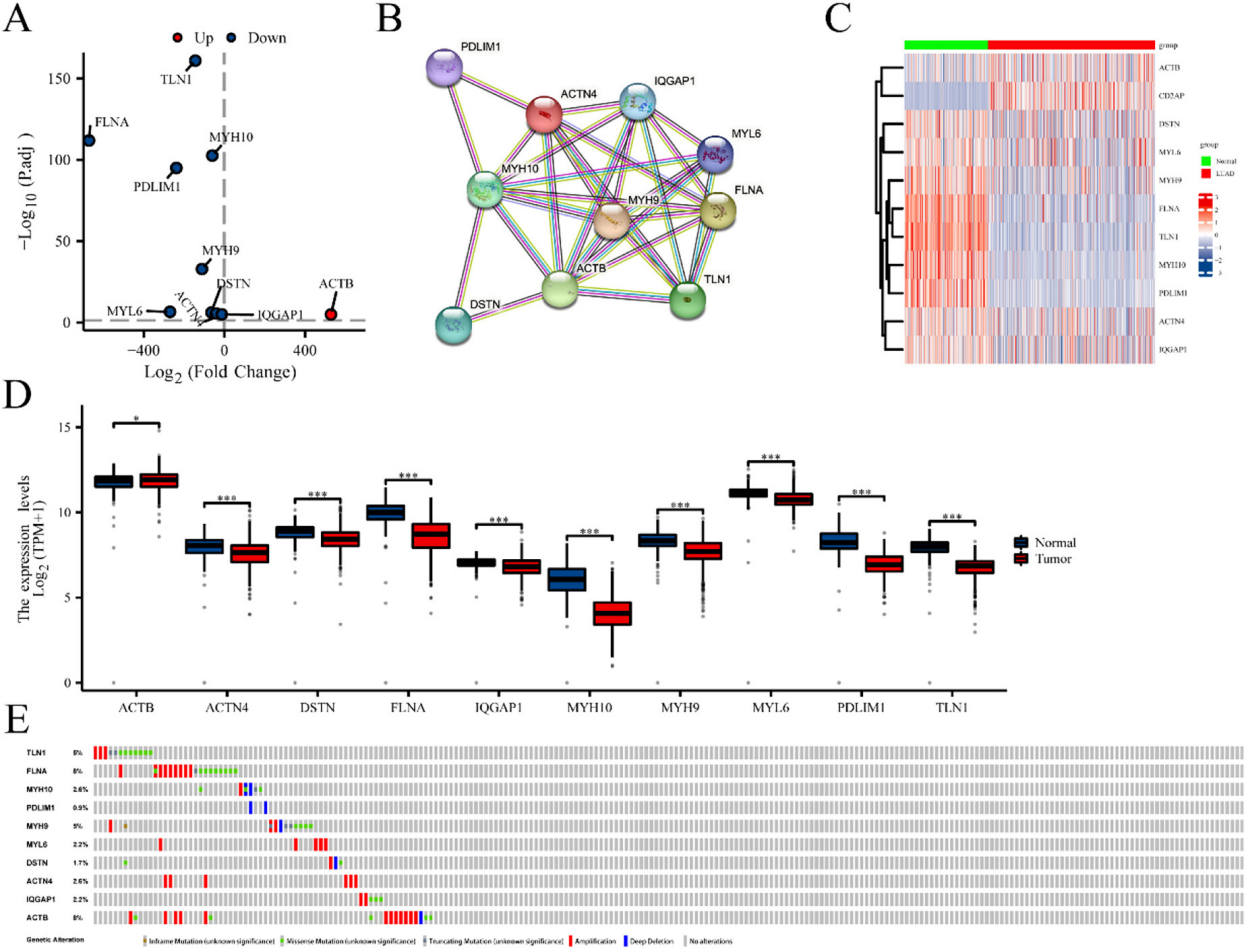
The differential expression of disulfidptosis-related genes (DRGs) in lung adenocarcinoma (LUAD) tissues compared to normal tissues. (A) Volcano plot indicated DRGs. (B) Protein-protein interaction network illustrated the interactions among DRGs. Heat map (C) and boxplots (D) of DRGs in LUAD compared the expression of DRGs in LUAD and normal tissues. (E) Mutation analysis of differentially expressed DRGs in TCGA-LUAD. *P < 0.05, and ^***^P< 0.001.

Fifteen genes (*ACTB*, *ACTN4*, *CAPZB*, *CD2AP*, *DSTN*, *FLNA*, *FLNB*, *INF2*, *IQGAP1*, *MYH10*, *MYH9*, *MYL6*, *PDLIM1*, *SLC7A11* and *TLN1*) associated with disulfidptosis were cataloged [11,12]. Differential expression of ten genes was identified from 15 DRGs using the R package “DESeq2” ( | log_2_FC  >  2, FDR  <  0.05). In LUAD, volcano plots, heat maps, and boxplots indicated that nine genes (*TLN1*, *FLNA*, *MYH10*, *MYH9*, *PDLIM1*, *DSTN*, *MYL6*, *ACTN4*, *IQGAP1*) were significantly downregulated, while *ACTB* was upregulated (Fig. 1A, C, D). The PPI network of these genes was depicted in Fig. 1B, and numerous mutations in DRGs were observed in LUAD (Fig. 1E).

**Figure 2.**
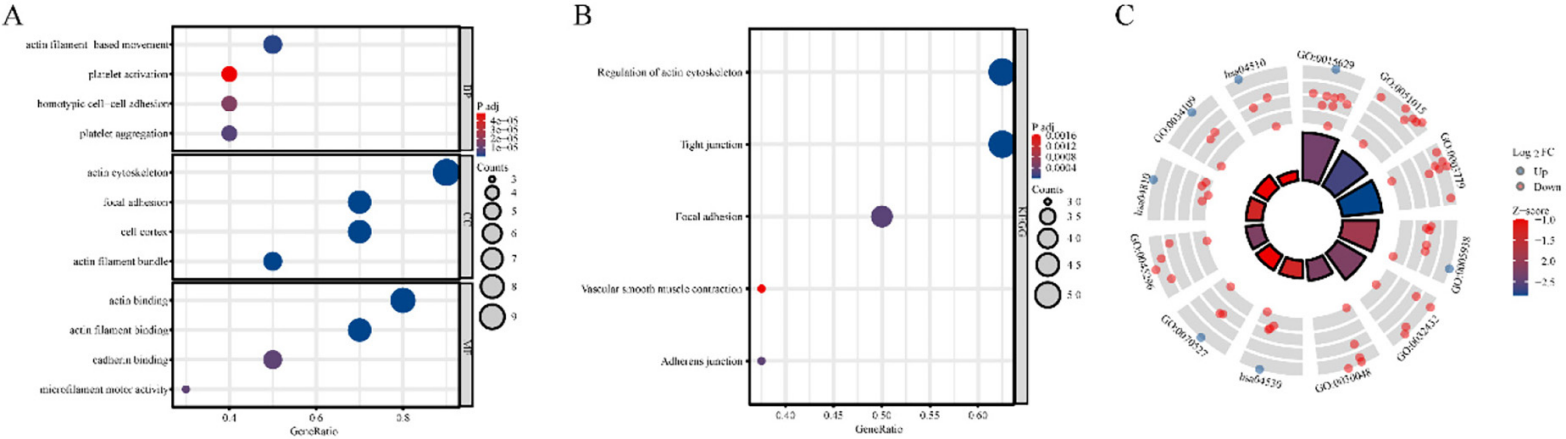
Functional Annotation of disulfidptosis-related genes (DRGs) in TCGA-LUAD: Enrichment analyses in GO (A) and KEGG (B–C) pathways.

**Figure 3.**
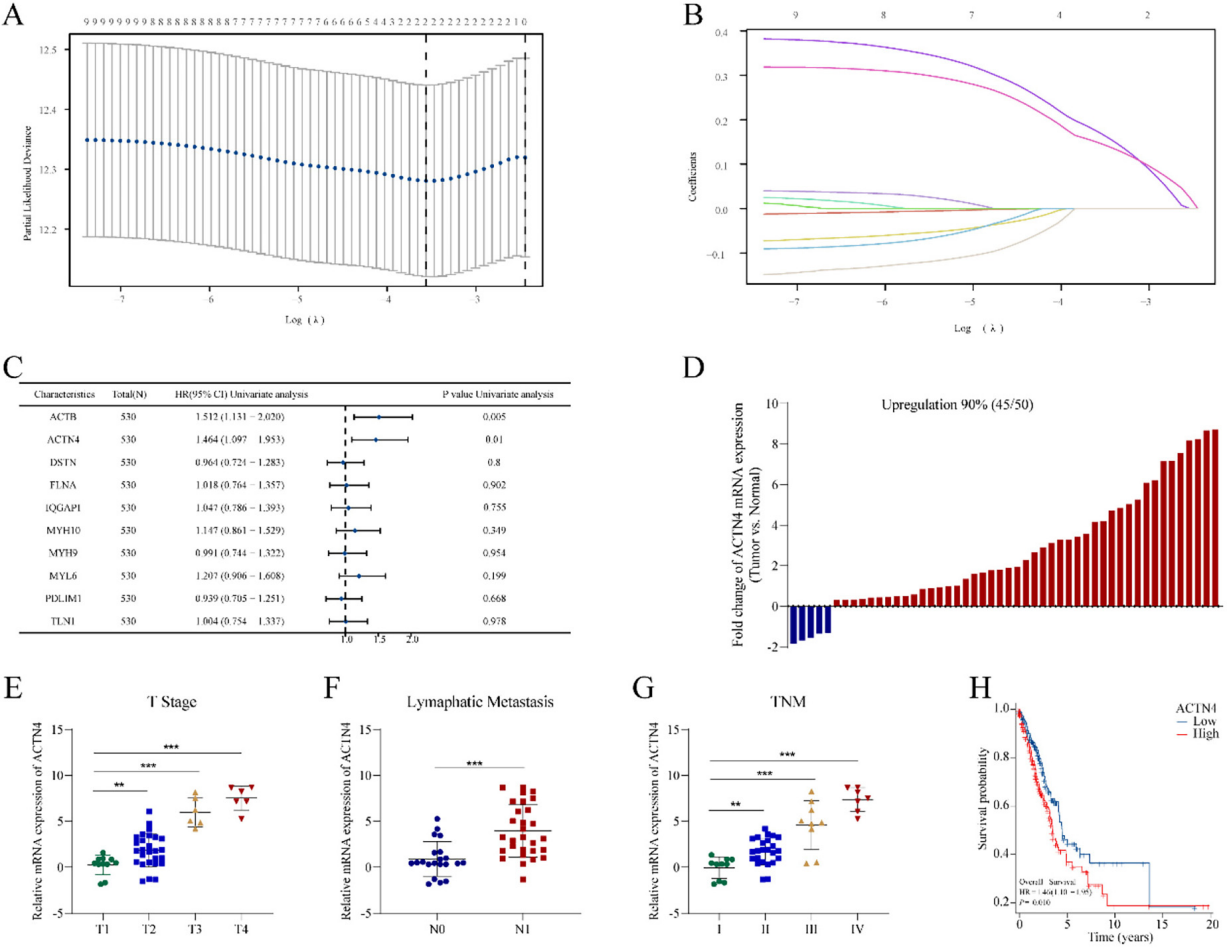
Construction of a risk prognostic model based on disulfidptosis-related genes (DRGs) in TCGA-LUAD. (A) LASSO regression of DRGs. (B) Cross-validation for tuning the parameter selection in the LASSO regression. (C) Univariate Cox regression analysis of DRGs. (D) The mRNA expression of *ACTN4* is upregulated in 90% of 50 lung adenocarcinoma (LUAD) tissues compared to normal tissues. The mRNA expression of *ACTN4* was positively with (E) T stage (^**^P< 0.01), (F) lymphatic metastasis (^**^P< 0.001), and (G) TNM stage (^**^P < 0.01). (H) LUAD patients with high expression of *ACTN4* have a lower percentage of overall survival. ^**^P< 0.01, and ^***^P< 0.001.

To enhance the understanding of the functions of DRGs that exhibit differential expression, enrichment analyses were conducted on GO and KEGG pathways. The outcomes of the enrichment analyses indicated that these genes were predominantly associated with cytoskeletal components and cell-generated adhesion factors (Fig. 2A–C).

**Table 1.** Correlation between ACTN4 expression and clinicalpathological characteristics in TCGA-LUAD.

Characteristics	Low expression of ACTN4	High expression of ACTN4	*P* value
*n*	269	270	
Age, *n* (%)			0.85995
⩽65	130 (25%)	127 (24.4%)	
> 65	131 (25.2%)	132 (25.4%)	
Gender, *n* (%)			0.57664
Female	141 (26.2%)	148 (27.5%)	
Male	128 (23.7%)	122 (22.6%)	
Smoker, *n* (%)			0.18059
No	33 (6.3%)	44 (8.4%)	
Yes	229 (43.6%)	219 (41.7%)	
T stage, *n* (%)			**0.04156**
T1	97 (18.1%)	79 (14.7%)	
T2	147 (27.4%)	145 (27.1%)	
T3	16 (3%)	33 (6.2%)	
T4	8 (1.5%)	11 (2.1%)	
N stage, *n* (%)			0.05105
N0	187 (35.8%)	163 (31.2%)	
N1	42 (8%)	55 (10.5%)	
N2&N3	31 (5.9%)	45 (8.6%)	
M stage, *n* (%)			0.30129
M0	185 (47.4%)	180 (46.2%)	
M1	10 (2.6%)	15 (3.8%)	
TNM stage, *n* (%)			**0.01221**
Stage I	166 (31.3%)	130 (24.5%)	
Stage II	53 (10%)	72 (13.6%)	
Stage III	34 (6.4%)	50 (9.4%)	
Stage IV	11 (2.1%)	15 (2.8%)	

**Figure 4.**
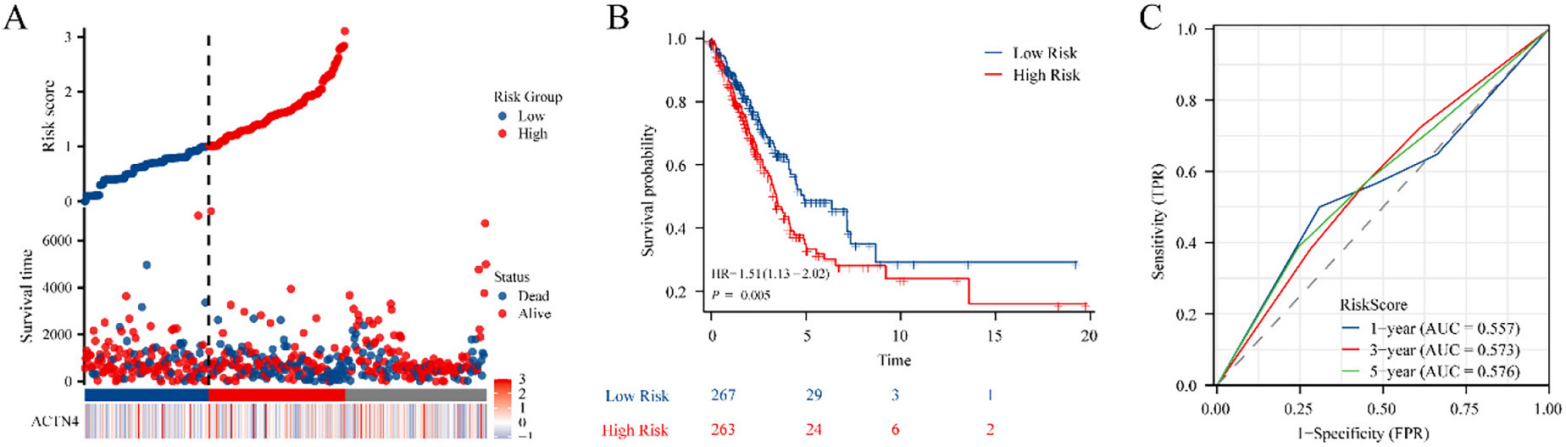
Construction of the prognostic model based on *ACTN4* and riskscore in TCGA-LUAD. (A) distribution of risk score, survival status and the expression of prognostic *ACTN4*. (B) Kaplan-Meier plot of the riskscore and overall survival. (C) ROCs for 1-year, 3-year and 5-year survival prediction.

### 3.2. Construction of a prognostic model based on DRGs

A prognostic model was developed utilizing 10 DRGs through LASSO regression, as depicted in Fig. 3A–B. Subsequently, two genes (*ACTB* and *ACTN4*) (*P* <  0.05) were identified as potential risk factors through univariate Cox regression analysis (Fig. 3C). Since *ACTB* is highly conserved and stably expressed housekeeping gene, it is not conducive to further research. Therefore, our focus was directed towards exploring the underlying mechanism of A*CTN4*. Furthermore, the chi-square test revealed a significant association between *ACTN4* levels and both T stage (*P* =  0.04156) and TNM stage (*P* =  0.01221) (Table 1). Comparing 50 pairs of LUAD and normal tissues, A*CTN4* mRNA expression was increased in 90% (55/60) of LUAD as compared to normal tissues (Fig. 3D), and was positively correlated with T stage (Fig. 3E), lymphatic metastasis (Fig. 3F), and TNM stage (Fig. 3G). The results of the survival analysis indicated that patients with elevated levels of *ACTN4* expression experienced a significantly lower overall survival rate (*P* =  0.010, Fig. 3H).

**Figure 5.**
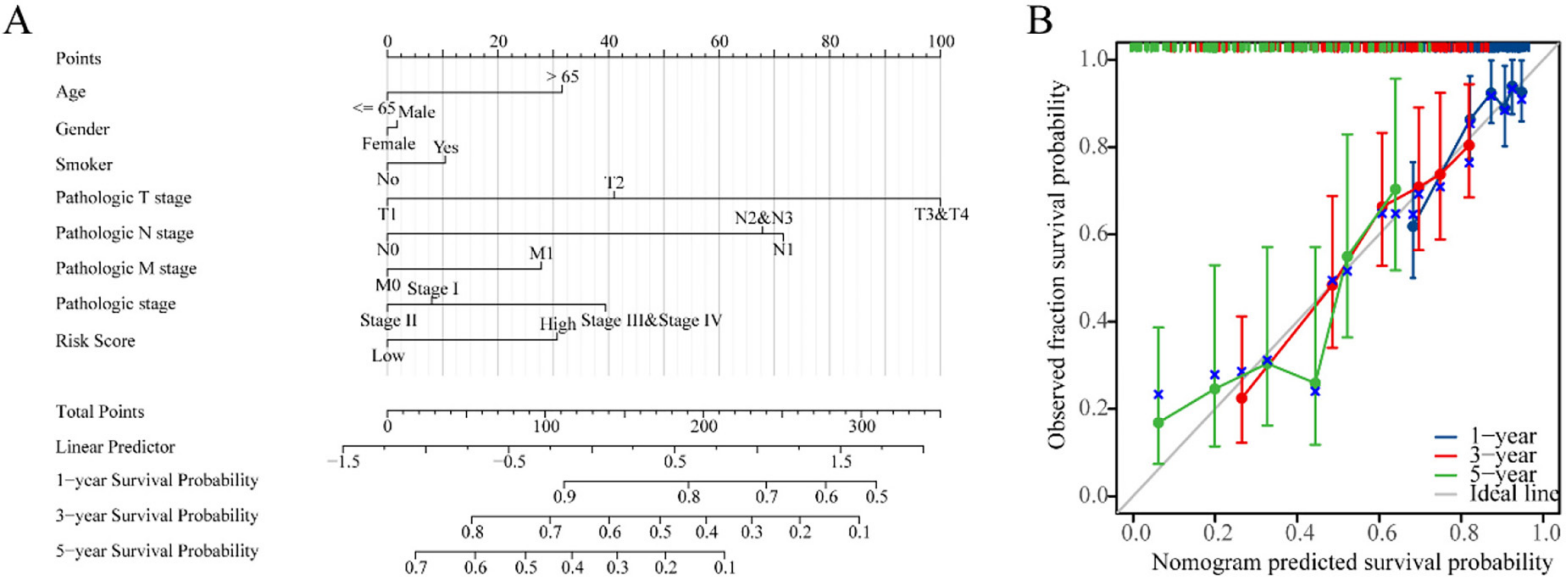
Nomogram development and validation. (A) Nomogram to predict the 1-year, 3-year and 5-year overall survival (OS) rate of lung adenocarcinoma (LUAD) patients. (B) Calibration curve for the OS nomogram model in LUAD.

### 3.3. Construction of the prognostic model based on ACTN4 and Riskscore

To explore the potential of *ACTN4* as a prognostic marker for LUAD, a prognostic model was constructed utilizing *ACTN4* expression and riskscore. The findings revealed that the cohort identified as high-risk demonstrated a significantly higher mortality rate and a decreased duration of survival when compared to the low-risk cohort. Furthermore, elevated scores were indicative of an unfavorable prognosis in LUAD (Fig. 4A). The Kaplan Meier curve provided additional evidence that patients categorized in the high-risk group exhibited an unfavorable prognosis (*P* =  0.005, Fig. 4B). Furthermore, time-dependent ROC analysis indicated that the prognostic precision of OS was 0.557 at 1 year, 0.573 at 3 years, and 0.576 at 5 years (Fig. 4C). The results indicated that the genetic signature of *ACTN4* may have practical implications for predicting the prognosis of LUAD.

### 3.4. Construction of nomogram and calibration curves

In order to improve the quantitative prognostication of LUAD, we developed a nomogram that integrates age, sex, smoking, T, N, M, stage and riskscore (Fig. 5A). Additionally, we generated a calibration curve that demonstrated a close alignment between the predicted and actual survival outcomes of LUAD patients (Fig. 5B). These results indicated that the inclusion of a risk score is a dependable method for forecasting the overall survival of individuals who have been diagnosed with LUAD.

### 3.5. Knockdown of ACTN4 inhibited LUAD cell proliferation

**Figure 6.**
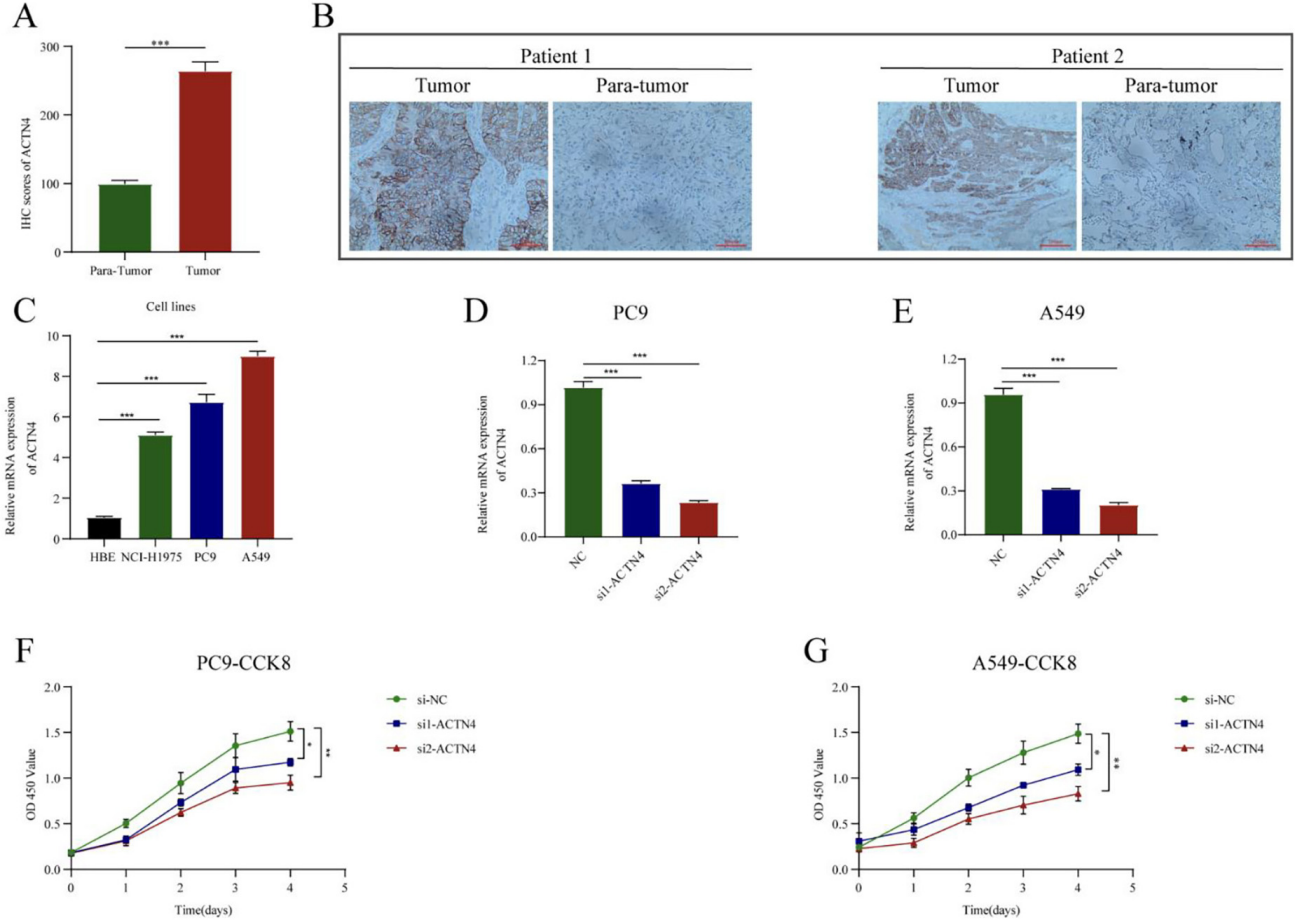
Knockdown of *ACTN4* inhibited lung adenocarcinoma (LUAD) cell proliferation. (A) The *ACTN4* staining score was up-regulated compared with that in adjacent normal tissues (*n* =  50). (B) Representative IHC staining images. (C) The mRNA expression of *ACTN4* in LUAD cell lines is higher than normal lung epithelial cells (HBE). (D-E) Two specific ACTN4-targeting siRNA significantly depleted the mRNAs of *ACTN4* in PC9 and A549 cells. (F-G) In CCK-8 assay, knockdown of *ACTN4* inhibited PC9 and A549 cell proliferation. *P< 0.05, ^**^P < 0.01, and ^***^P< 0.001.

IHC staining showed that *ACTN4* was significantly higher than in adjacent normal tissues compared to LUAD cancer tissues (*n* =  50, Fig. 6A). Additionally, IHC images of two patients were presented in Fig. 6B. Furthermore, *ACTN4* expression was significantly higher in LUAD cell lines compared to HBE. To further elucidate the biological function of *ACTN4* in LUAD, two specific *ACTN4*-targeting siRNA were transfected into A549 and PC9 cells (Fig. 6D–E). CCK8 proliferation assay indicated that the knockdown of *ACTN4* expression inhibited the proliferation in LUAD cells (Fig. 6F–G).

### 3.6. ACTN4 expression is associated with immune signatures in LUAD

**Figure 7.**
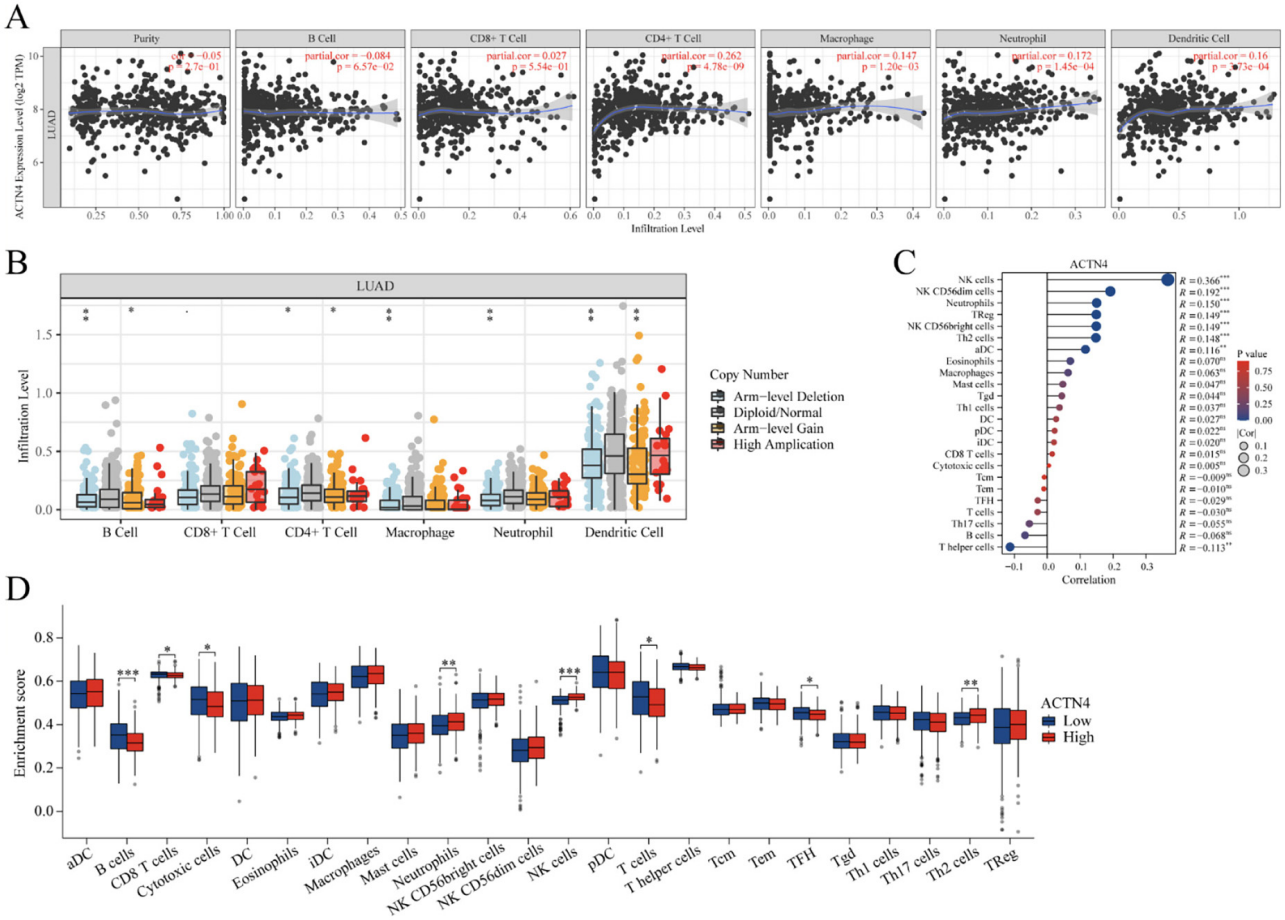
Correlations of *ACTN4* expression with immune infiltration level in lung adenocarcinoma (LUAD). (A) The expression of *ACTN4* was significantly correlated with infiltrating levels of CD4+ T cells, macrophages, neutrophil and dendrites cells in LUAD. (B) *ACTN4* CNV affects the infiltrating levels of B cells, CD4+ T cells, macrophages, neutrophils, and dendritic cells in LUAD. (C) The change ratio of 22 immune cell subtypes in the high and low *ACTN4* expression groups in LUAD. *P< 0.05, ^**^P < 0.01, and ^***^P < 0.001. (D) ssGSEA algorithm calculated the differential expression of 24 immune cell markers in high and low ACTN4 expression groups.

The existence of tumor-infiltrating lymphocytes is an autonomous predictor for both lymph node invasion and survival [28]. As depicted in Fig. 7A, TIMER2 website analysis indicated that *ACTN4* expression was correlated with CD4+ T cells (*P* =  4.78 × 10^−9^), macrophages (*P* =  1.20 × 10^−3^), neutrophil (*P* =  1.45 × 10^−4^) and dendrites cells (*P* =  3.73 × 10^−4^), suggesting a pivotal role of *ACTN4* in the immune infiltration of LUAD. Furthermore, a significant correlation was observed between *ACTN4* CNV and the level of infiltration by B cells, CD4+ T cells, macrophages, neutrophils, and dendritic cells (Fig. 7B). The ssGSEA algorithm was used to calculate the markers of 24 immune cells to verify the immune infiltration of *ACTN4* in LUAD (Fig. 7C).

**Table 2.** Correlation analysis between *ACTN4* and relate genes and markers of immune cells in TIMER.

Gene markers	Gene markers	partial.cor	*P* value
B cell	CD19	−0.105890	1.87E-02*
	CD20	−0.120400	7.44E-03^**^
	CD70	0.082183	6.83E-02
CD8+ T Cell	CD8A	0.041848	3.54E-01
	CD8B	0.000887	9.84E-01
	CD25	0.040319	3.72E-01
Tfh	CD183	0.125097	5.41E-03^**^
	CD185	0.013307	7.68E-01
	CD278	−0.049711	2.71E-01
Th1	CD212	0.106543	1.80E-02*
	CD191	0.081040	7.22E-02
	CD195	0.129645	3.93E-03^**^
Th2	CD194	−0.052490	2.45E-01
	CD198	0.043452	3.36E-01
	CD365	0.031213	4.89E-01
Th17	CD360	0.103248	2.19E-02*
	IL23R	−0.035306	4.34E-01
	CD196	−0.061000	1.76E-01
Treg	FOXP3	0.141300	1.66E-03^**^
	CD73	0.217631	1.07E-06^***^
	CD127	0.025895	5.66E-01
T cell exhaustion	PD-1	0.120094	7.60E-03^**^
	CTLA4	−0.026046	5.64E-01
	LAG3	0.149673	8.57E-04^***^
Macrophage	CD68	0.161359	3.22E-04^***^
	CD11b	0.197407	1.01E-05^***^
M1 macrophage	NOS2	0.126003	5.08E-03^**^
	IRF5	0.181242	5.18E-05^***^
M2 macrophage	CD163	0.150301	8.15E-04^***^
	CD206	0.042607	3.45E-01
TAM	CCL2	0.116461	9.65E-03^**^
	CD86	0.161359	3.22E-04^***^
Monocyte	CD14	0.105238	1.94E-02*
	CD33	0.013952	7.57E-01
Natural killer cell	CD57	0.081677	7.00E-02
	KIR3DL1	0.073553	1.03E-01
	CD7	0.125883	5.12E-03^**^
Neutrophl	CD16	0.114612	1.09E-02*
	CD55	−0.123207	6.16E-03^**^
Dendritic cell	CD1C	−0.116498	9.63E-03^**^
	CD141	0.165256	2.28E-04^***^

In order to examine the relationship between *ACTN4* and a range of immune infiltrating cells in LUAD, the TIMER tool was employed to assess the correlation of *ACTN4* with multiple immune cell markers in LUAD (Table 2). The findings indicated a noteworthy correlation between the expression of *ACTN4* and the expression of B cell immune markers *CD20* and *CD19* (*P* <  0.05, Table 2). A diverse array of T cells with varying functions were analyzed. Upon controlling for tumor purity, a noteworthy association was observed between the expression of *ACTN4* and specific immune markers of T cells, including *CD183*, *CD212*, *CD195*, *CD360*, *FOXP3*, *CD73*, *PD-1*, and *LAG3* (*P* <  0.05, Table 2). This observation implies that *ACTN4* could potentially participate in the T cell immune response to LUAD. Furthermore, a noteworthy association was observed between the expression level of *ACTN4* and the immune markers *NOS2* and *IRF5* of M1 macrophages, implying that *ACTN4* may exert regulatory control over macrophages (*P* <  0.05, Table 2). Additionally, we uncovered a noteworthy association between *ACTN4* and the immune markers *CD68*, *CD11b*, *CD163*, *CCL2*, *CD86*, *CD14* and *CD7* of NK cells, neutrophils, and dendritic cells in LUAD (*P* <  0.05, Table 2), signifying that the expression of *ACTN4* in LUAD is intricately connected to the infiltration of immune cells through diverse mechanisms.

Furthermore, 539 LUAD samples were classified into two distinct groups based on their *ACTN4* expression levels (high = 270, low = 269). To investigate the potential variances in the tumor immune microenvironment between the two groups, we conducted an analysis of the differential expression of 22 immune cells (Fig. 7D). Our findings revealed that the *ACTN4* high expression group demonstrated an increase in neutrophils, NK cells, and Th2 cells (*P* <  0.05), while B cells, CD8+ T cells, cytotoxic cells, T cells, and TFH were observed to be decreased in comparison to the low expression group (*P* <  0.05).

### 3.7. ACTN4 Expression is Associated with m6A RNA Methylation Regulators in LUAD

**Figure 8.**
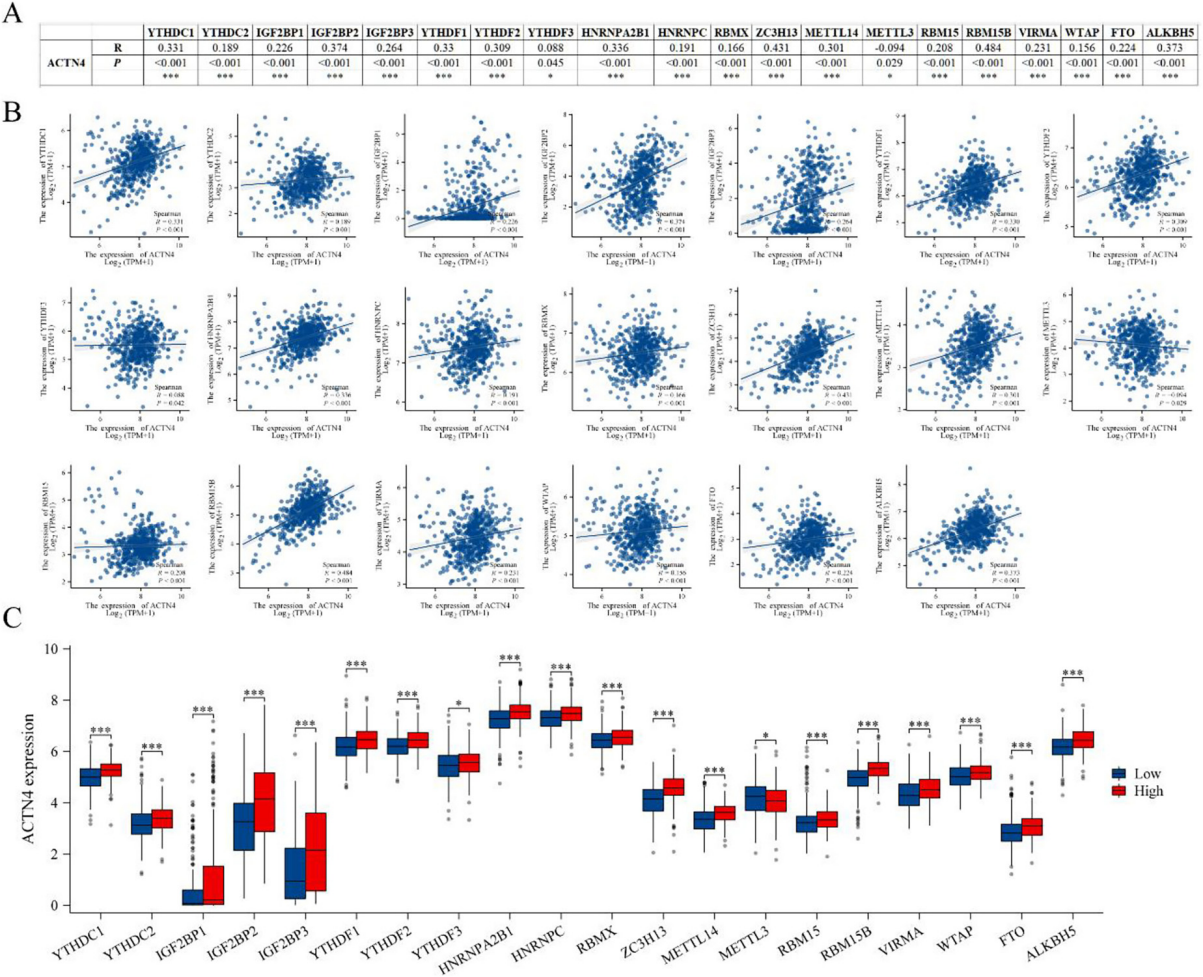
Correlations of *ACTN4* expression with m6A related genes in lung adenocarcinoma (LUAD). (A) TCGA-LUAD analyzed the correlation between the expression level of *ACTN4* and m6A-related genes. (B) Draw a scatter plot to show the correlation between *ACTN4* and m6A related genes. (C) The differential expression of m6A related genes in the high and low *ACTN4* expression groups in LUAD. *P < 0.05, and ^***^P < 0.001.

The acknowledged importance of m6A modification in the progression of LUAD prompted our analysis of the expression correlation between *ACTN4* and 20 m6A-related genes in the TCGA LUAD dataset [25,29]. Our findings indicate a significant correlation between *ACTN4* expression and the 20 m6A-related genes in LUAD (*P* <  0.05, Fig. 8A–B). Furthermore, we found that the expression of m6a related genes was significantly higher in the high-expression *ACTN4* group compared to the low-expression group (*P* <  0.05, Fig. 8C), with the exception of *METTL3* which demonstrated a decrease (*P* <  0.05, Fig. 8C). These outcomes imply a close association between *ACTN4* and m6A modification in LUAD.

## 4. Discussion

LUAD is a prevalent and perilous neoplasm characterized by a high incidence of metastasis and mortality, and represents a significant area of interest for cancer research [1]. To effectively address this disease and elucidate its underlying mechanisms, a more profound understanding is necessary. Recent investigations have uncovered a novel mode of cell death, known as disulfidptosis, which has been implicated in cancer-related pathways [9,12]. Disulfide has been shown to mitigate cellular oxidative stress by activating the antioxidant enzyme system, thereby impeding tumorigenesis and progression [30]. For example, tumor cells have the ability to manipulate the intracellular redox environment by utilizing disulfides, which facilitates the growth and dissemination of tumors [31]. Antineoplastic agents, including cisplatin and paclitaxel, hinder tumor initiation and progression by interacting with intracellular disulfides [32,33]. Consequently, the concept of disulfidptosis presents a promising opportunity for the advancement of therapeutic interventions for LUAD.

The aim of this study was to construct a prognostic model that employs DRGs to diagnose and forecast the prognosis of patients with LUAD. Initially, potential risk genes were identified through the use of univariate Cox and Lasso Cox regression analyses, with *ACTN4* being singled out. Subsequently, a prognostic model was developed utilizing the expression of *ACTN4* and riskscore, which exhibited effectiveness in prognosticating the outcome of LUAD. As a result, experimental investigations were conducted to elucidate the precise role of *ACTN4*. IHC analysis revealed that the expression of *ACTN4* protein in LUAD was higher than in adjacent tissues, and its expression was correlated with clinicopathological features. *In vitro* cell experiments demonstrated that the inhibition of *ACTN4* expression could suppress the proliferation of LUAD cells, which is in accordance with previous studies [34].

*ACTN4* expression level was significantly correlated with a variety of immune cells and immune cell marker genes. Notably, high expression of *ACTN4* was positively correlated with increased levels of neutrophils, NK cells, and Th2 cells, while the levels of B cells, CD8+ T cells, cytotoxic cells, T cells, and TFH were observed to be reduced. We speculate that *ACTN4* has the potential to augment and fortify the immune response of neoplastic cells through the modulation of memory B cells, which are capable of identifying and binding tumor-specific antigens and generating tumor-specific antibodies. Memory B cells have the ability to collaborate with various immune cells, including T cells, macrophages, and dendritic cells, to enhance the activity and effectiveness of the immune response, resulting in a more robust attack on malignant cells due to their extended persistence in the body [35]. These findings suggest that *ACTN4* plays a crucial role in the immune infiltration of LUAD. As a result, it is posited that an excess of *ACTN4 in vivo* would prompt immune reactions against tumors. Nevertheless, further controlled and clinical trials are required to validate this hypothesis.

The presence of M6A modification in eukaryotic RNA is evident in numerous biological processes, particularly in the promotion of tumor development within the tumor immune microenvironment [36]. The current investigation has demonstrated a noteworthy association between the expression of *ACTN4* and 20 m6A-related genes. Furthermore, the heightened expression of *ACTN4* has been linked to elevated levels of 19 m6A-related genes, except for *METTL3* which displayed a reduction. These observations imply that *ACTN4* may undergo m6A modification, thereby enhancing mRNA stability and ultimately influencing the tumor immune microenvironment, thereby facilitating tumor progression.

Disulfidptosis, a novel form of cell death, was identified by Liu et al. [9]. They observed that sugar-deficient cancer cells with high expression of *SLC7A11* experienced disrupted disulfide binding between cytoskeletal proteins due to the accumulation of disulfide substances, leading to histone skeleton breakdown and cell death. *SLC7A11*, a key determinant of glucose deficiency-induced cell death in various cancer cell lines [37,38], is frequently overexpressed in multiple cancers, often correlating with poor patient outcomes [39,40,41]. Studies have shown that *SLC7A11* knockout mice exhibit no distinct phenotype in major organs, indicating its potential as a promising therapeutic target in cancer treatment [42]. Recent research has focused on exploiting *SLC7A11*-associated metabolic vulnerabilities (such as glucose or glutamine dependence) to induce disulfidptosis, offering new strategies for cancer therapy [43]. Liu et al. [44] demonstrated that compared to cancer cells with low *SLC7A11* expression, inhibitors of glucose transporters (*GLUT*) were more effective in inducing disulfidptosis in cells with high *SLC7A11* expression. The use of KL-11743, a potent inhibitor of GLUT1 and *GLUT3*, selectively suppressed the growth of *SLC7A11*-expressing tumors in cell line xenografts and lung cancer patient-derived xenografts [45]. Furthermore, high expression of *SLC7A11* can serve as a biomarker for selecting cancer patients who may benefit from glutaminase or *GLUT* inhibitors for disulfidptosis treatment, offering a new avenue for targeting cancer metabolism weaknesses through metabolic therapy.

However, there are some limitations to our study. This was a retrospective study, with data from public databases lacking information such as treatment and relapse records. Our conclusions need to be validated in vivo or in vitro experiments and prospective clinical studies.

## 5. Conclusions

In brief, the current study has developed a novel prognostic model utilizing DRGs, which demonstrates a high degree of efficacy in predicting the prognosis of patients with LUAD. Furthermore, *in vitro* experiments have revealed a significant upregulation of *ACTN4* in LUAD, which is closely associated with the clinicopathological features of patients and can facilitate the proliferation of LUAD cells. Additionally, a comprehensive analysis has been conducted to investigate the correlation between *ACTN4* expression and tumor immune infiltration as well as m6A modification. The expression of *ACTN4* displays a strong association with various immune cells, thereby potentially impeding the infiltration of memory B cells and influencing the immune response against tumors. The m6A-mediated modification of *ACTN4* mRNA may augment its stability, consequently impacting the tumor immune microenvironment and facilitating the progression of LUAD.
